# Risk Factors for Severe COVID-19 Outcomes Among Persons Aged ≥18 Years Who Completed a Primary COVID-19 Vaccination Series — 465 Health Care Facilities, United States, December 2020–October 2021

**DOI:** 10.15585/mmwr.mm7101a4

**Published:** 2022-01-07

**Authors:** Christina Yek, Sarah Warner, Jennifer L. Wiltz, Junfeng Sun, Stacey Adjei, Alex Mancera, Benjamin J. Silk, Adi V. Gundlapalli, Aaron M. Harris, Tegan K. Boehmer, Sameer S. Kadri

**Affiliations:** ^1^Clinical Epidemiology Section, Critical Care Medicine Department, National Institutes of Health Clinical Center, Bethesda, Maryland; ^2^Laboratory of Malaria and Vector Research, National Institute of Allergy and Infectious Diseases, Rockville, Maryland; ^3^CDC COVID-19 Response Team.

Vaccination against SARS-CoV-2, the virus that causes COVID-19, is highly effective at preventing COVID-19–associated hospitalization and death; however, some vaccinated persons might develop COVID-19 with severe outcomes^†^ (*1**,**2*). Using data from 465 facilities in a large U.S. health care database, this study assessed the frequency of and risk factors for developing a severe COVID-19 outcome after completing a primary COVID-19 vaccination series (primary vaccination), defined as receipt of 2 doses of an mRNA vaccine (BNT162b2 [Pfizer-BioNTech] or mRNA-1273 [Moderna]) or a single dose of JNJ-78436735 [Janssen (Johnson & Johnson)] ≥14 days before illness onset. Severe COVID-19 outcomes were defined as hospitalization with a diagnosis of acute respiratory failure, need for noninvasive ventilation (NIV), admission to an intensive care unit (ICU) including all persons requiring invasive mechanical ventilation, or death (including discharge to hospice). Among 1,228,664 persons who completed primary vaccination during December 2020–October 2021, a total of 2,246 (18.0 per 10,000 vaccinated persons) developed COVID-19 and 189 (1.5 per 10,000) had a severe outcome, including 36 who died (0.3 deaths per 10,000). Risk for severe outcomes was higher among persons who were aged ≥65 years, were immunosuppressed, or had at least one of six other underlying conditions. All persons with severe outcomes had at least one of these risk factors, and 77.8% of those who died had four or more risk factors. Severe COVID-19 outcomes after primary vaccination are rare; however, vaccinated persons who are aged ≥65 years, are immunosuppressed, or have other underlying conditions might be at increased risk. These persons should receive targeted interventions including chronic disease management, precautions to reduce exposure, additional primary and booster vaccine doses, and effective pharmaceutical therapy as indicated to reduce risk for severe COVID-19 outcomes. Increasing COVID-19 vaccination coverage is a public health priority.

Data from 465 facilities in the Premier Healthcare Database Special COVID-19 Release (PHD-SR) were analyzed.^§^ Persons who completed primary vaccination (including those who might have received additional doses as part of their primary vaccination series, and booster vaccine doses) were included in the analysis.^¶^ Persons with partial vaccination recorded in PHD-SR were excluded. COVID-19 was identified by querying all encounters in PHD-SR during March 2020–October 2021.** Severe outcomes were defined as any one of the following: diagnosis of acute respiratory failure, need for NIV, ICU admission, or death.^ ††^ The risk for COVID-19 severe outcomes and deaths per 10,000 persons were calculated among persons who completed primary vaccination. Among persons with COVID-19 after primary vaccination, a logistic regression model was specified to estimate the odds for severe versus nonsevere outcomes. Covariates included age group, sex, race/ethnicity, six selected underlying conditions (Supplementary Table, https://stacks.cdc.gov/view/cdc/113043),^§§^ vaccine type, time since primary vaccination, prevalence of the SARS-CoV-2 B.1.617.2 (Delta) variant,^¶¶^ and previous COVID-19 (defined as COVID-19 occurring >90 days before primary vaccination). Receipt of anti–SARS-CoV-2 monoclonal antibodies*** was not entered in the model, because no severe outcomes were identified in this subgroup. Statistically significant risk factors for severe outcomes were identified from the model, and the number of risk factors per person was calculated. All analyses were performed using SAS statistical software (version 9.4; SAS Institute); p-values <0.05 were considered statistically significant. This activity was reviewed by CDC and conducted consistent with applicable federal law and CDC policy.^†††^

During December 2020–October 2021, a total of 1,228,664 persons aged ≥18 years completed primary vaccination (Pfizer-BioNTech, 72.8%; Moderna, 20.0%, Janssen, 6.5%; unspecified mRNA vaccine, 0.8%) across 465 facilities in PHD-SR. Among these, 2,246 (18 per 10,000) acquired COVID-19, including 327 who were hospitalized, 189 (1.5 per 10,000) who had a severe COVID-19 outcome, and 36 (0.3 per 10,000) who had a COVID-19–related death (including nine persons discharged to hospice). Among those who acquired COVID-19 after primary vaccination, 1.6% (36) died, 1.1% (24) survived and were admitted to an ICU, and 5.7% (129) survived and received a diagnosis of acute respiratory failure or required NIV but were not admitted to an ICU (Table). 

**TABLE:** Characteristics of persons with COVID-19 after completing a primary COVID-19 vaccination series, overall and by disease outcome, and adjusted odds ratios for severe COVID-19 outcomes — 465 health care facilities, United States, December 2020–October 2021

Characteristic	No. (%) with COVID-19 after primary vaccination			aOR of severe versus nonsevere COVID-19 outcome (95% CI)
	Total (N = 2,246)	Nonsevere outcome (n = 2,057)	Severe outcome (n = 189)	
**Disposition**				
Outpatient	1,360 (60.6)	NA	NA	NA
ED/Observation	559 (24.9)	NA	NA	NA
Hospitalization	327 (14.6)	NA	NA	NA
**Severe outcome**				
Any severe outcome	189 (8.4)	NA	NA	NA
Death	36 (1.6)	NA	NA	NA
Survivors admitted to ICU*	24 (1.1)	NA	NA	NA
Survivors with respiratory failure,^†^ without ICU admission or death	129 (5.7)	NA	NA	NA
**Sex**				
Female	1,294 (57.6)	1,204 (58.6)	90 (47.6)	1.0
Male	951 (42.3)	852 (41.4)	99 (52.4)	1.17 (0.95–1.44)
Unknown	1 (0.0)	1 (0.0)	0 (—)	NA
**Age group, yrs**				
18–39	251 (11.2)	248 (12.1)	3 (1.6)	1.0
40–64	807 (35.9)	772 (37.5)	35 (18.5)	1.52 (0.82–2.83)
≥65	1,188 (52.9)	1,037 (50.4)	151 (79.9)	3.22 (1.81–5.74)
**Race/Ethnicity**				
Hispanic	77 (3.4)	75 (3.6)	2 (1.1)	0.47 (0.18–1.19)
Asian, non-Hispanic	52 (2.3)	49 (2.4)	3 (1.6)	0.86 (0.33–2.24)
Black, non-Hispanic	323 (14.4)	288 (14.0)	35 (18.5)	1.25 (0.92–1.69)
White, non-Hispanic	1,643 (73.2)	1,502 (73.1)	141 (74.6)	1.0
Other, non-Hispanic	87 (3.9)	80 (3.9)	7 (3.7)	0.88 (0.47–1.66)
Unknown	64 (2.8)	63 (3.1)	1 (0.5)	0.37 (0.11–1.18)
**Vaccine type**				
Janssen (Johnson & Johnson)	196 (8.8)	173 (8.4)	23 (12.2)	1.0
Moderna	422 (18.9)	397 (19.3)	25 (13.2)	0.56 (0.32–0.98)
Pfizer-BioNTech	1,618 (72.4)	1,479 (71.9)	139 (73.5)	0.70 (0.39–1.26)
Unspecified mRNA vaccine	10 (0.4)	8 (0.4)	2 (1.1)	1.19 (0.15–9.74)
**Days since primary vaccination series completion**				
≤60	325 (13.3)	290 (14.1)	35 (18.5)	1.0
61–120	409 (18.2)	377 (18.3)	32 (16.9)	0.93 (0.62–1.41)
>120	1,512 (67.3)	1,390 (67.6)	122 (64.6)	0.72 (0.41–1.27)
**Infected during Delta variant^†,§^ predominance**	1,819 (81.0)	1,676 (81.5)	143 (75.7)	1.36 (0.82–2.25)
**Underlying medical conditions**				
Overweight/Obesity	609 (27.1)	532 (25.9)	77 (40.7)	1.28 (0.97–1.7)
Diabetes mellitus	633 (28.2)	535 (26.0)	98 (51.9)	1.47 (1.14–1.89)
Immunosuppression	446 (19.9)	360 (17.5)	86 (45.5)	1.91 (1.37–2.66)
Chronic kidney disease	353 (15.7)	271 (13.2)	82 (43.4)	1.61 (1.19–2.19)
Chronic neurologic disease	301 (13.4)	242 (11.8)	59 (31.2)	1.54 (1.06–2.25)
Chronic cardiac disease	753 (33.5)	624 (30.4)	129 (68.3)	1.44 (1.01–2.06)
Chronic pulmonary disease	889 (39.6)	752 (36.6)	137 (72.5)	1.69 (1.31–2.18)
Chronic liver disease	124 (5.5)	103 (5.0)	21 (11.1)	1.68 (1.12–2.52)
**Previous COVID-19 illness**	68 (3.0)	67 (3.3)	1 (0.5)	0.27 (0.09–0.84)
**Receipt of monoclonal antibody therapy**	446 (19.9)	446 (21.7)	0 (—)	NA
**Receipt of booster/additional vaccine doses**	27 (1.2)	24 (1.2)	3 (1.6)	NA
**At least one risk factor^¶^**	1,728 (76.9)	1,539 (74.8)	189 (100)	NA

**Abbreviations:** aOR = adjusted odds ratio; ED = emergency department; ICU = intensive care unit; NA = not applicable.

* Survivors admitted to ICU include all survivors requiring invasive mechanical ventilation.

^†^ Respiratory failure defined as diagnostic coding for acute respiratory failure or need for noninvasive ventilation.

^§^ SARS-CoV-2 B.1.617.2 (Delta) variant.

**^¶^** Risk factors for severe COVID-19 outcomes after completion of a primary vaccination series, identified in the multivariable model (age ≥65 years, diabetes mellitus, immunosuppression, chronic kidney disease, chronic liver disease, chronic neurologic disease, chronic cardiac disease, or chronic pulmonary disease).

Adjusted odds ratios (aOR) of severe COVID-19 outcomes after primary vaccination were higher among persons aged ≥65 years (aOR = 3.22; 95% CI = 1.81–5.74), and those with immunosuppression (aOR = 1.91; 95% CI = 1.37–2.66), pulmonary disease (aOR = 1.69; 95% CI = 1.31–2.18), liver disease (aOR = 1.68; 95% CI = 1.12–2.52), chronic kidney disease (aOR = 1.61; 95% CI = 1.19–2.19), neurologic disease (aOR = 1.54; 95% CI = 1.06–2.25), diabetes (aOR = 1.47; 95% CI = 1.14–1.89), or cardiac disease (aOR = 1.44; 95% CI = 1.01–2.06) (Figure 1). Compared with persons who received the Janssen vaccine, Pfizer-BioNTech recipients had similar odds of severe outcomes (aOR = 0.70; 95% CI = 0.39–1.26), whereas recipients of the Moderna vaccine had lower odds (aOR = 0.56; 95% CI = 0.32–0.98). Odds of severe outcomes did not differ significantly by sex, race/ethnicity, time since primary vaccination, or whether infection occurred during the period of Delta variant predominance. Previous COVID-19 illness was associated with reduced odds of severe outcomes (aOR = 0.27; 95% CI = 0.09–0.84).

**FIGURE 1:**
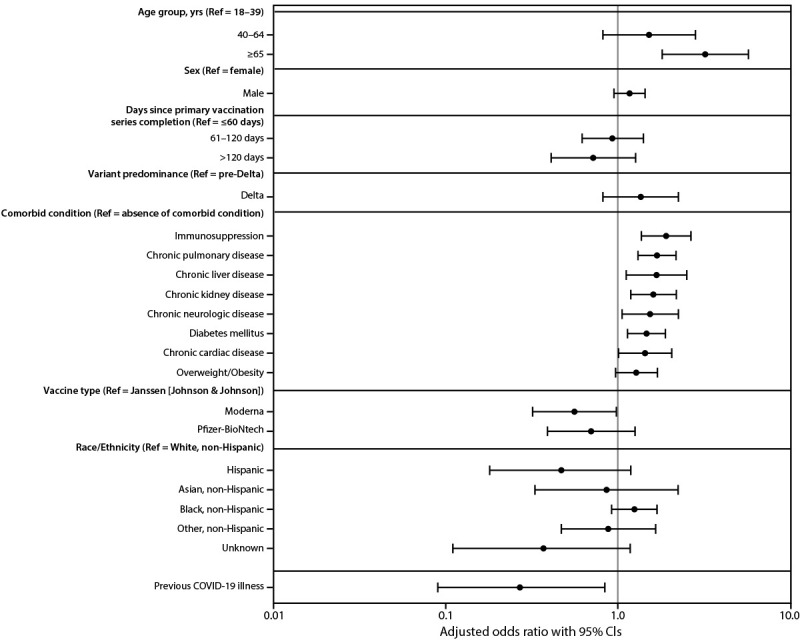
Risk factors for severe COVID-19 among persons who completed a primary COVID-19 vaccination series — 465 health care facilities, United States, December 2020–October 2021 **Abbreviations: **Delta = SARS-CoV-2 B.1.617.2 (Delta) variant; Ref = referent group.

Among 446 persons with COVID-19 after primary vaccination who received anti–SARS-CoV-2 monoclonal therapy (casirivimab and imdevimab [93.3%] or bamlanivimab and etesivimab [6.7%]), none experienced severe outcomes. Among 3,395 persons who received booster or additional vaccine doses, 27 (0.8%) acquired COVID-19, three of whom experienced severe outcomes (but no ICU admissions or deaths).

All persons with severe COVID-19 outcomes after primary vaccination had at least one of the eight risk factors identified as significant in the model. The frequency of having four or more risk factors increased with disease severity, ranging from 18.8% (386) among persons who had nonsevere outcomes, 56.9% (87) among survivors who had respiratory failure or were admitted to an ICU, to 77.8% (28) among persons who died. Among 36 persons who died, 15 (41.7%) had do-not-resuscitate orders at the time of hospital admission (Figure 2).

**FIGURE 2:**
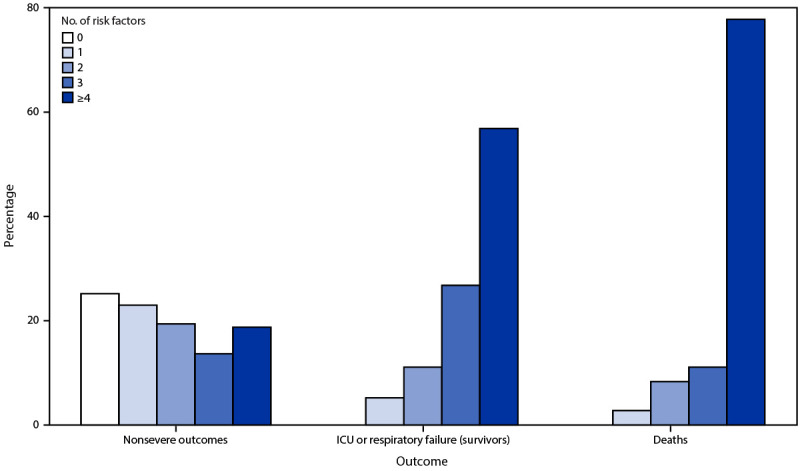
Frequency of risk factors in persons with COVID-19 after completion of a primary vaccination series, by outcome*,† — 465 health care facilities, United States, December 2020–October 2021 **Abbreviation: **ICU = intensive care unit. * Outcome totals: nonsevere = 2,057; ICU/respiratory failure = 153; deaths = 36. ^^†^^ All persons in the ICU or respiratory failure (survivors) and deceased groups had at least one risk factor.

## Discussion

In this analysis of data from 465 U.S. health care facilities, severe COVID-19 outcomes (i.e., respiratory failure, ICU admission, or death) were rare among adults aged ≥18 years after primary vaccination. These findings are consistent with studies that have shown that COVID-19 vaccination lowers the likelihood of COVID-19–associated hospitalization and death (*1**,**2*). Risk for a severe COVID-19 outcome after primary vaccination was higher among persons aged ≥65 years, were immunosuppressed, or had one of six other underlying conditions; all persons with severe COVID-19 outcomes after primary vaccination had at least one risk factor. This study provides insight into the frequency of and risk factors for severe outcomes among persons who acquired COVID-19 after primary vaccination during periods of pre-Delta and Delta variant predominance; findings might not be applicable to the risk from SARS-CoV-2 B.1.1.529 (Omicron) variant or future variants.

In this study, age ≥65 years, immunosuppression, diabetes, and chronic kidney, cardiac, pulmonary, neurologic, and liver disease were associated with higher odds for severe COVID-19 outcomes;^§§§^ all persons with severe COVID-19 outcomes after primary vaccination had at least one of these risk factors. These findings are consistent with those of previous studies of a largely prevaccination U.S. population (*3*) and a U.K. population predominantly vaccinated with ChAdOx1-SARS-COV-2 (AstraZeneca) vaccine (*4*). Approximately one half of U.S. adults have a major chronic disease that increases their risk for severe COVID-19 (*5*). Even after primary vaccination, a significant proportion of the population might remain at risk and require additional strategies to prevent severe COVID-19 outcomes.

Population-wide data have demonstrated that COVID-19 hospitalization and death are more frequent among Hispanic, non-Hispanic Black, and non-Hispanic American Indian or Alaska Native persons than among non-Hispanic White persons.^¶¶¶^ This might be explained by higher levels of SARS-CoV-2 exposure, reduced access to care, and higher rates of uncontrolled underlying conditions experienced by these populations (*6*); however, this study did not find an association between race/ethnicity and severe COVID-19 outcomes after primary vaccination, suggesting that COVID-19 vaccines are important for helping to mitigate racial and ethnic disparities exacerbated by the COVID-19 pandemic.

Several factors could contribute to severe outcomes in populations who are at risk, including suboptimal response to vaccination, waning immunity, and predisposition to severe disease. Persons who might not have mounted a protective immune response after initial vaccination might benefit from an additional primary dose (*2*). Booster vaccination after primary vaccination has been demonstrated to further reduce the risk for infection, particularly severe COVID-19 (*7*), and is recommended by CDC for all persons aged ≥18 years.**** Pharmaceutical therapies are also available for preventing and treating COVID-19 in at-risk populations.^††††^ In addition, findings from this study complement data from clinical trials (*8**,**9*) suggesting that anti–SARS-CoV-2 monoclonal antibodies when appropriate might protect vaccinated persons with COVID-19 from experiencing severe outcomes.

The findings in this report are subject to at least five limitations. First, the reliance on procedure, diagnosis, and billing codes to define vaccination status, underlying conditions, and outcomes might have led to misclassification because of inaccurate or incomplete records. In addition, presence of underlying conditions might not be fully collected by administrative coding. Second, outcomes that occurred during COVID-19 encounters might have been related to other factors (e.g., diminished access to routine services for control of chronic diseases might have exacerbated severe outcomes in persons with comorbidities). Third, the components of the composite outcome are not necessarily of equal severity and results should be interpreted accordingly; the number of deaths alone was too small to allow analysis of risk factors in this subgroup. Fourth, persons with underlying conditions might be more likely to access health care, thereby disproportionately increasing COVID-19 risk estimates in this group compared with persons without underlying conditions. Finally, PHD-SR represents a convenience sample of health care facilities, limiting generalizability to the U.S. population.

Approximately 70% of eligible adults in the United States have completed a primary COVID-19 vaccination series.^§§§§^ With the emergence of novel variants of concern and development of additional therapeutic strategies, studies in vaccinated populations are vital to guide targeted guidelines and interventions for persons at risk for severe outcomes. COVID-19–associated outcomes occurred in a small proportion of persons (0.015%) who had completed primary vaccination, all of whom were aged ≥65 years, immunosuppressed, or had other underlying conditions. Even when vaccinated, persons with identifiable risk factors should receive interventions including chronic disease management, precautions to reduce exposure, additional primary and booster vaccine doses, and effective pharmaceutical therapy as indicated to reduce risk for severe COVID-19–associated outcomes. Increasing COVID-19 vaccination coverage is a public health priority.

**Summary**

**What is already known about this topic?**

COVID-19 vaccines are highly effective against COVID-19–associated hospitalization and death.

**What is added by this report?**

Among 1,228,664 persons who completed primary vaccination during December 2020–October 2021, severe COVID-19–associated outcomes (0.015%) or death (0.0033%) were rare. Risk factors for severe outcomes included age ≥65 years, immunosuppressed, and six other underlying conditions. All persons with severe outcomes had at least one risk factor; 78% of persons who died had at least four.

**What are the implications for public health practice?**

Vaccinated persons who are older, immunosuppressed, or have other underlying conditions should receive targeted interventions including chronic disease management, precautions to reduce exposure, additional primary and booster vaccine doses, and effective pharmaceutical therapy to mitigate risk for severe outcomes. Increasing vaccination coverage is a critical public health priority.

## Footnotes

†
https://www.medrxiv.org/content/10.1101/2021.07.08.21259776v1

§ PHD-SR, formerly known as the PHD COVID-19 Database, is a large U.S. health care all-payor administrative database that includes inpatient and hospital-based outpatient (e.g., emergency department or clinic) health care encounters from >900 geographically diverse, nonprofit, nongovernmental, community, and teaching hospitals and health systems from rural and urban areas. PHD-SR represents approximately 20% of U.S. inpatient admissions. Of all reporting centers, 465 reported vaccination data during December 2020–October 2021 and were included in the study. Updated PHD-SR data are released every 2 weeks; data for this report were obtained from PHD-SR release date November 8, 2021. https://offers.premierinc.com/rs/381-NBB-525/images/PHD_COVID-19_White_Paper.pdf

¶ Completion of a primary vaccination series was defined as receipt of the second of 2 doses of an mRNA vaccination series (Pfizer-BioNTech or Moderna]) or a single dose of Janssen ≥14 days before onset of illness. Only vaccines with Food and Drug Administration (FDA) full or emergency use authorization were considered in this definition. The definition of persons who completed primary vaccination included persons who might have received either or both of additional doses as part of their primary vaccination series or booster vaccine doses after primary vaccination (1.2% received additional vaccine doses). Vaccination was collected using current procedural terminology (CPT) codes (0001A and 002A for first and second Pfizer-BioNTech doses, respectively, 0011A and 0012A for first and second Moderna doses, respectively, 0031A for Janssen vaccine) and standard charge codes (510771000010000 and 510771000020000 for first and second Pfizer-BioNTech doses, respectively, 510771000110000 and 510771000120000 for first and second Moderna doses, respectively, and 510771000310000 for Janssen vaccine).

** Inpatient and outpatient encounters for COVID-19 were identified based on primary or secondary diagnosis coding for COVID-19 (legacy coding with *International Classification of Diseases, Tenth Revision, Clinical Modification* (ICD-10-CM) code B97.29 [other coronavirus as the cause of diseases classified elsewhere] for March 2020, ICD-10-CM codes U07.1 [COVID-19] or J12.82 [pneumonia due to coronavirus disease 2019] during March 2020–October 2021), or SARS-CoV-2 reverse transcription–polymerase chain reaction (RT-PCR) positivity. Encounters were excluded if there had been a previous COVID-19 encounter within the 90 days preceding vaccination to minimize collection of readmissions of infections occurring before vaccination and/or persistent positive RT-PCR test results.

†† Acute respiratory failure was identified by ICD-10-CM codes (J80, J96.00, J96.90, R06.00, R06.03, R06.09, R06.3, R06.89, and R09.2) or procedure codes (5A1935Z, 5A1945Z, and 5A1955Z); NIV was identified using CPT code 94660 with exclusion of persons with concurrent ICD-10-CM coding for obstructive sleep apnea (G47.33) or obesity hypoventilation syndrome (E66.2) to avoid confounding because of chronic use; ICU admission was identified from hospital chargemaster records (a comprehensive list of items billable from an encounter); deaths were identified from discharge status. Deaths included persons discharged to hospice. Where a person had several COVID-19 encounters meeting criteria, outcomes were defined based on the encounter with the most severe outcome.

§§ Underlying medical conditions were identified based on ICD-10-CM coding present on any (one or more) inpatient or outpatient encounter in PHD-SR during January 2019–October 2021. Conditions were selected to encompass the majority of medical conditions identified by CDC as being associated with higher risk for severe COVID-19. https://www.cdc.gov/coronavirus/2019-ncov/science/science-briefs/underlying-evidence-table.html

¶¶ SARS-CoV-2 B.1.617.2 (Delta) variant predominance was defined as the period when the Delta variant accounted for ≥50% of sequenced isolates in the United States. Delta variant prevalence represented <50% of sequenced isolates before June 19, 2021 and ≥50% of sequenced isolates from June 20, 2021 onwards, per CDC genomic surveillance data (https://covid.cdc.gov/covid-data-tracker/#variant-proportions) (Accessed December 12, 2021). PHD-SR provides monthly temporal resolution (rather than weekly or daily) for encounter data; therefore, the end of June 2021 was used as the cutoff for pre-Delta and Delta variant predominant phases (pre-Delta = March 2020–June 2021; Delta = July 2021–October 2021).

*** Receipt of anti-SARS-CoV-2 monoclonal antibodies with FDA approval under emergency use authorization for treatment of COVID-19 (bamlanivimab and etesevimab; casirivimab and imdevimab; and sotrovimab) were collected using standard charge codes entered during COVID-19 encounters (250250132240000, 250250132310000, 250250132770000, 250250132780000, 250250132790000, 250250132800000, 250250133110000, 250250133130000, 250250133600000, 250250133730000, 250888027880000, 250888028180000, 250888028190000, 510771002390000, 510771002400000, 510771002410000, 510771002430000, 510771002440000, 510771002450000, 510771002460000, 510771002470000, and 510771002480000). Two of these monoclonal preparations are currently approved for use in combination only (bamlanivimab/ etesevimab and casirivimab/ imdevimab); for these combinations, administration of the two components had to be documented within 24 hours of each other. Use of monoclonal antibodies for post- or preexposure prophylaxis was not evaluated in this study.

††† 45 C.F.R. part 46, 21 C.F.R. part 56; 42 U.S.C. Sect. 241(d); 5 U.S.C. Sect. 552a; 44 U.S.C. Sect. Sect. 3501 et seq.

§§§ CDC’s National Center for Chronic Disease Prevention and Health Promotion collects data on chronic disease in the U.S. population using a set of 124 indicators. https://www.cdc.gov/mmwr/pdf/rr/rr6401.pdf (Accessed December 12, 2021).

¶¶¶ CDC collects data on risk for SARS-CoV-2 infection, hospitalization, and death by race/ethnicity from COVID-NET, a population-based surveillance system collecting data through a network of 250 acute-care hospitals across 14 states. https://www.cdc.gov/coronavirus/2019-ncov/covid-data/investigations-discovery/hospitalization-death-by-race-ethnicity.html (Accessed December 12, 2021).

**** CDC recommends booster vaccination with any one of the three FDA-approved COVID-19 vaccines for all persons aged ≥18 years, 2 months or more after receiving the Janssen vaccine or 6 months or more after completing a primary mRNA vaccine series. In addition, persons aged 16–17 years can receive a Pfizer-BioNTech COVID-19 vaccine booster 6 months after completing their primary vaccination series. https://www.cdc.gov/coronavirus/2019-ncov/vaccines/booster-shot.html

††††
https://emergency.cdc.gov/han/2021/han00461.asp

§§§§ CDC collects data on vaccine delivery and administration in the United States and reports it through the CDC COVID Data Tracker. https://covid.cdc.gov/covid-data-tracker/#vaccinations_vacc-total-admin-rate-total (Accessed December 12, 2021).
